# The Effect of PPAR*α*, PPAR*δ*, PPAR*γ*, and PPARpan Agonists on Body Weight, Body Mass, and Serum Lipid Profiles in Diet-Induced Obese AKR/J Mice

**DOI:** 10.1155/2007/97125

**Published:** 2007-06-06

**Authors:** W. Wallace Harrington, Christy S. Britt, Joan G. Wilson, Naphtali O. Milliken, Jane G. Binz, David C. Lobe, William R. Oliver, Michael C. Lewis, Diane M. Ignar

**Affiliations:** Department of Metabolic Diseases, GlaxoSmithKline Research, Research Triangle Park, NC 27709, USA

## Abstract

Activation of peroxisome proliferator-activated receptor (PPAR) *α*, *δ*, and *γ* subtypes increases expression of genes involved in fatty acid transport and oxidation and alters adiposity in animal models of obesity and type-2 diabetes. PPARpan agonists which activate all three receptor subtypes have antidiabetic activity in animal models without the weight gain associated with selective PPAR*γ* agonists. Herein we report the effects of selective PPAR agonists (GW9578, a PPAR*α* agonist, GW0742, a PPAR*δ* agonist, GW7845, a PPAR*γ* agonist), combination of PPAR*α* and *δ* agonists, and PPARpan (PPAR*α*/*γ*/*δ*) activators (GW4148 or GW9135) on body weight (BW), body composition, food consumption, fatty acid oxidation, and serum chemistry of diet-induced obese AKR/J mice. PPAR*α* or PPAR*δ* agonist treatment induced a slight decrease in fat mass (FM) while a PPAR*γ* agonist increased BW and FM commensurate with increased food consumption. The reduction in BW and food intake after cotreatment with PPAR*α* and *δ* agonists appeared to be synergistic. GW4148, a PPARpan agonist, induced a significant and sustained reduction in BW and FM similar to an efficacious dose of rimonabant, an antiobesity compound. GW9135, a PPARpan agonist with weak activity at PPAR*δ*, induced weight loss initially followed by rebound weight gain reaching vehicle control levels by the end of the experiment. We conclude that PPAR*α* and PPAR*δ* activations are critical to effective weight loss induction. These results suggest that the PPARpan compounds may be expected to maintain the beneficial insulin sensitization effects of a PPAR*γ* agonist while either maintaining weight or producing weight loss.

## 1. INTRODUCTION

Obesity has risen to epidemic proportions world wide and is one the most visible, yet often neglected, of public health issues. It is now prevalent in virtually all age and socio-economic groups in both developed and developing nations [1]. Obesity is a complex, multifactorial condition produced by genetic, social, and psychological factors, the most significant being high-fat diet and sedentary life style. The health consequences of obesity range from increased risk of premature death to serious chronic conditions such as type 2 diabetes, dyslipidemia, atherosclerosis, hypertension, cardiovascular diseases, stroke, and certain forms of cancer [2–5]. Agents that reduce obesity through reductions in food intake or increased energy expenditure could serve as therapeutic options for the prevention of obesity and its comorbidities [6–8]. 

Peroxisome proliferator-activated receptors (PPARs) are ligand-activated transcription factors that belong to the superfamily of nuclear receptors [9]. Three subtypes, designated PPAR*α* (NR1C1), PPAR*δ* (NR1C2), and PPAR*γ* (NR1C3) have been identified whose endogenous ligands include fatty acids and fatty acid metabolites. PPARs form heterodimers with retinoid X receptors (RXRs) and bind to the hexanucleotidic PPAR responsive element (PPRE), thereby regulating the expression of target genes involved in lipid and carbohydrate metabolism.

PPARs are found in species ranging from Xenopus to humans [9] with each receptor having a distinct tissue expression profile. PPAR*α* is expressed mainly in the liver, heart, and muscle. The discovery that fibrates are hypolipidemic agents which activate PPAR*α* suggested that this receptor may play a role in lipid metabolism [9, 10]. Indeed, activation of PPAR*α* has been shown to upregulate genes involved in hepatic lipid and lipoprotein metabolism and fatty acid oxidation in skeletal muscle. In addition, these agents decrease adiposity in animal models of obesity and type-2 diabetes mellitus (T2DM). For example, fenofibrate has been shown to reduce food intake, body weight, and adiposity in several mouse models and obesity-prone rats [11, 12]. PPAR*δ* has a broad pattern of distribution and is expressed in many tissues, including muscle and kidney [13]. Recent work has suggested that PPAR*δ* is involved in overall energy regulation and fatty acid oxidation in the muscle. Activation of PPAR*δ* has also been shown to increase high-density lipoprotein cholesterol (HDL-c) in diabetic db/db mice and obese rhesus monkeys [14]. Studies by Wang et al. [15] suggest that overexpression of PPAR*δ* in adipose tissue protects against diet-induced obesity in mice and treatment with a PPAR*δ* selective agonist reduces weight gain without effects on food intake in fat-fed mice [16].

The discovery that glitazones activate PPAR*γ* receptor has elucidated the role of this receptor in lipid transport and storage and carbohydrate metabolism [17]. PPAR*γ* is expressed predominantly in white and brown adipose tissue and is important in the regulation and control of adipocyte development and function [18]. Treatment with PPAR*γ* agonists enhances the action of insulin and reduces serum glucose in subjects with T2DM, however, substantial body weight gain also occurs that is comprised of both fat mass and fluid volume [19–22]. 

PPARpan agonists can activate all three PPAR receptor subtypes and exert a variety of effects on multiple tissues simultaneously. This class of compounds has been shown to have antidiabetic efficacy in several animal models of T2DM [23]. These compounds also affect lipoprotein composition and reduce atherosclerotic plaque formation without the weight gain associated with PPAR*γ* agonists suggesting their utility in treatment of metabolic syndrome [24, 25]. 

A number of studies have described the effect of individual PPAR agonists in a variety of animal models or experimental paradigms [14, 26–28]. This study provides a systematic four-week evaluation of potent and selective agonists of the three PPAR isoforms, the combination of PPAR*α* and *δ* agonists and PPARpan agonists in a single chronic model of diet-induced obesity. We report the effects of these agents on body weight, body composition, fatty acid oxidation, and clinical chemistry in obesity-prone AKR/J mice. 

## 2. METHODS

### 2.1. In vitro potency and selectivity

#### 2.1.1. Assessment of PPAR activation using GAL4 transient transfection assay

The functional potency of selected ligands was evaluated using a transient transfection assay in CV-1 cells. The ligand binding domains for murine PPAR*α*, PPAR*δ*, and PPAR*γ* were fused to the yeast transcription factor GAL4 DNA binding domain as a chimera. CV-1 cells were propagated and transiently transfected with expression vectors for the respective PPAR chimera as previously described [29, 30]. Test compounds were compared to reference comparators that give maximum responses in this assay. Compounds which produced an activation of at 70% or greater, compared to a positive control, were considered full agonists.

#### 2.1.2. Ex vivo quantification of PPAR-induced fatty acid oxidation

Fatty acid oxidation (FAO) was determined by ^14^C-labeled CO_2_ capture from tissue homogenates using a method modified from Dohm et al. [31]. Following treatment with either vehicle or a PPAR agonist, livers from fed mice were surgically removed and a section excised from the same lobe. The tissue was immediately weighed, minced with scissors and placed in tubes (Falcon #2063) on ice. Cold SET buffer (250 mM Sucrose, 1 mM EDTA, 10 mM Tris, pH 7.4) was added at a ratio of 10 mL SET:1 gram of tissue and the tissue homogenized on ice for 15 sec using a hand-held homogenizer (Polytron PT1200; Kinematica AG). The homogenates remained on ice until assayed.

The labeled reaction buffer was prepared by first drying ^14^C-oleic acid (0.5 *μ*Ci/reaction; PerkinElmer #NEC-317) under nitrogen. The dried fraction is re-suspended in unlabeled oleic acid such that the final concentration of oleic acid in the reaction buffer was 0.2 mM. BSA was added slowly while mixing to a final concentration of 0.5% and the mixture was incubated at 37°C for 15 minutes. The labeled cocktail was then added to the reaction buffer to give a produce concentration of 100 mM sucrose, 10 mM Tris pH 7.4, 4 mM ATP, 0.05 mM Coenzyme A, 0.1 mM malic acid, 1 mM magnesium chloride, 80 mM potassium chloride, 5 mM potassium phosphate, 0.2 mM EDTA, and 2 mM L-carnitine, as described previously [32, 33].

Oxidation reactions were performed in tubes (Falcon #352059) fitted with a stopper top (KONTES Glass Co., #882310-0000), center well (KONTES #882320-0000), and filter (Socorex #322.02) soaked with 175 *μ*L of 1N NaOH. 100 *μ*L of homogenate was dispensed into each tube and the reactions initiated by adding 400 *μ*L of reaction buffer. The tubes were quickly capped and incubated with gentle shaking for 60 minutes in a 37°C water bath. After incubation, the filters were removed, from the tubes, placed in 7 mL of scintillant, and counted for 2 minutes (PerkinElmer Tri-Carb 3100TR). The oxidative activity of each compound was calculated as nmole CO_2_ captured/gram tissue/hour and reported as fold change relative to vehicle control.

### 2.2. In vivo animal studies

All procedures were performed in compliance with the Animal Welfare Act, USDA regulations and approved by the GlaxoSmithKline Institutional Animal Care and Use Committee. Animals were housed at 72°F and 50% relative humidity with a 12-hour light and dark cycle.

### 2.2.1. Compounds

All compounds evaluated were synthesized by the Medicinal Chemistry Department at GlaxoSmithKline, Inc., and were determined to be >90% pure by HPLC and/or NMR analysis [34]. Dosing solutions of GW7845, GW0742, GW9578, GW4148, and GW9135 were prepared as a suspension in a vehicle of 0.5% methylcellulose and 0.1% Tween 80 and dosed at 10 mL/kg. Doses of each PPAR ligand were chosen from results of previous in-house efficacy studies.

### 2.2.2. Effect of PPAR agonists on body weight, body mass, and food consumption

The effects of monotherapy with selective PPAR agonists, combination therapy with PPAR*α* and PPAR*δ*, and treatment with PPARpan agonists were evaluated in four experiments in diet-induced obese (DIO) AKR/J mouse. The AKR/J mouse is a polyoma-susceptible strain originally utilized to study accelerated tumor development [35]. This strain becomes obese and hyperinsulemic when fed a high fat diet [36–39]. Age-matched, male AKR/J mice were allowed ad libitum access to Research Diet D12331 (Research Diet, Brunswick, NJ) at the Jackson Laboratories (Bar Harbor, ME) beginning at 6 weeks of age. The diet has an energy density of 5.56 kcal/g (58% kcal from fat; 26% kcal from carbohydrates, and 16% kcal from protein). The animals were allowed to become obese, achieving BW >40 grams before shipping to GlaxoSmithKline laboratory animal facility at 13 weeks of age. The mice were housed 4 per cage in standard shoebox cages and were fed the high fat diet until they reached approximately 50 grams. Age-matched lean control animals obtained from Jackson Laboratories were fed a diet of normal chow (3.04 kcal/g energy density, 12% kcal from fat, LabDiet 5001, St. Louis, MO) and used for comparison.

At the beginning of each study, the animals were weighed and body composition obtained using an EchoMRI-100 quantitative magnetic resonance (qMR, EchoMRI, Houston, TX) whole body composition analyzer [40, 41]. Mice were sorted into groups (*n* = 8–10/group) such that BW and body mass (% lean and fat mass) were not significantly different at the beginning of the study. 16 lean control mice on standard chow were used as reference. All mice were dosed orally with vehicle (0.5% methylcellulose and 0.1% Tween 80, 10 mL/kg) for six days prior to the beginning of dosing for acclimation to handling and treatment before drug treatment was initiated.

In each experiment, BW of each animal was measured and recorded three times weekly throughout the treatment period. Body mass was obtained weekly on days 0, 7, 13, 20, and 27 of treatment. The effects of selective PPAR*α*, *δ*, and *γ* agonists on food consumption were also assessed. Food consumption is expressed as total energy consumed (kcal) over a 24-period and as cumulative consumption over the course of the experiment.

The fat content of Research Diets D12331 chow results in pellets that crumbles making it difficult to quantify food consumption thus, Research Diets D12451 chow (4.7 kcal/g (45% kcal from fat, 35% kcal from carbohydrates and 20% kcal from protein)) was used in studies where food consumption was determined as these pellets are more solid. The animals were transitioned two weeks before compound dosing from Research Diets D12331 chow to Research Diets D12451 chow. Previous experiments (data not presented) have shown that animals fed this diet maintain the same BW and fat mass level as observed at the time of transition.

On the final day of each experiment, a terminal blood sample (800–1000 *μ*L) was obtained via cardiac puncture under isoflurane anesthesia. Whole blood was placed in a Terumo Capiject blood collection tube (Terumo Medical Corp., Elkton, Md, USA), allowed to sit at room temperature for 20 minutes then centrifuged to obtain serum. Serum levels of glucose, triglycerides, glycerol, nonesterified fatty acids, total cholesterol, the high-density lipoprotein cholesterol, and *β*-hydroxybutyrate were determined in all mice using an Olympus AU640 clinical chemistry immuno-analyzer (Olympus America Inc., Melville, NY, USA). In addition, liver weights were obtained following the terminal blood sample on the final day of the study and samples were used to determine liver fatty acid oxidation activity.

## 3. EXPERIMENTAL DESIGN

Experiment 1 was designed to study the effects of a selective PPAR*α* agonist and PPAR*δ* agonist as mono and combination therapy. 48 mice were sorted into 6 groups and blocked such that initial BW and body composition were not different between groups. Three groups of animals (*n* = 8) were dosed with Vehicle, the PPAR*α* agonist (GW9578, 1 mg/kg), or the PPAR*δ* agonist (GW0742, 30 mg/kg) for 4 weeks. Two additional groups of mice were dosed for the first 14 days with either a PPAR*α* agonist or a PPAR*δ* agonist alone. At day 15, the PPAR*δ* agonist was added to the treatment regimen of animals dosed with PPAR*α*, and the PPAR*α* agonist was added to the dosing material of animals previously dosed with PPAR*δ* alone. The sixth group was dosed with both the PPAR*α* and PPAR*δ* agonists for the entire 28-day period. BW and food consumption were assessed 3 times per week and body composition was measured weekly.

In Experiment 2, 32 mice were sorted into 4 groups (*n* = 8/group) and dosed with vehicle and a selective PPAR*γ* agonist (GW7845, 3 mg/kg) for 28 days. Rimonabant (RIM, 10 or 30 mg/kg, q.d.), a CB1 receptor antagonist, was used as a positive control for weight loss. As in Experiment 1, BW and food consumption were determined 3 times per week and body composition was measured weekly.

In Experiment 3, three groups of mice (*n* = 9) were dosed for 28 days with vehicle or GW4148 (3 or 10 mg/kg), a PPARpan agonist that potently activates all three receptor subtypes. In Experiment 4, five groups of mice (*n* = 8) were dosed for 28 days with vehicle or GW9135 (3 or 10 mg/kg), a PPARpan agonist that has a different profile of PPAR*α*, *δ*, and *γ* activation from GW4148.

## 4. DATA ANALYSIS

All data are expressed as mean ± standard error of the mean. Weight loss experiments were analyzed using Analysis of Covariance (ANCOVA) with repeated measures followed by Dunnett's post hoc test. Comparison of serum chemistry values, food consumption and fat and lean mass changes between start and end of studies was analyzed by two-way analysis of variance with repeated measures model (ANOVA) followed by Dunnett's post hoc test. Values were considered to be significant when a value of *P* < .05 was achieved.

## 5. RESULTS

### 5.1. Assessment of PPAR activation using GAL4 transient transfection assay

Each compound evaluated in vivo was characterized with regard to activation of the three PPAR subtypes [36] as shown in Table 1. These compounds are full agonists of their respective receptors. GW9578 is a potent agonist of murine PPAR*α* receptors with an EC_50_ of 8 nM and more than a 250-fold selectivity over PPAR*γ* and PPAR*δ* [34]. GW0742 is a potent and selective PPAR*δ* agonist, (EC_50_ = 28 nM) having a 300-fold selectivity over PPAR*α* and PPAR*γ* [26]. GW7845 is a potent and selective PPAR*γ* agonist with an EC_50_ of 1.2 nM and >1000-fold selectivity over the other murine PPAR subtypes [27]. Both PPARpan agonists used in this study activate all of the PPAR subtypes, however, GW4148 and GW9135 have different potency profiles. GW4148 is nearly equipotent at murine PPAR*α*/*δ*/*γ* (EC_50_ < 100 nM), while GW9135 is most potent at the PPAR*α* receptor with significant activity on PPAR*γ* and weak potency at PPAR*δ*.

### 5.2. In vivo studies

*Experiment 1. Effect of mono- and combination therapy of PPAR*α* and PPAR*δ* Agonists in Obese AKR/J Mice*

**Figure 1:**
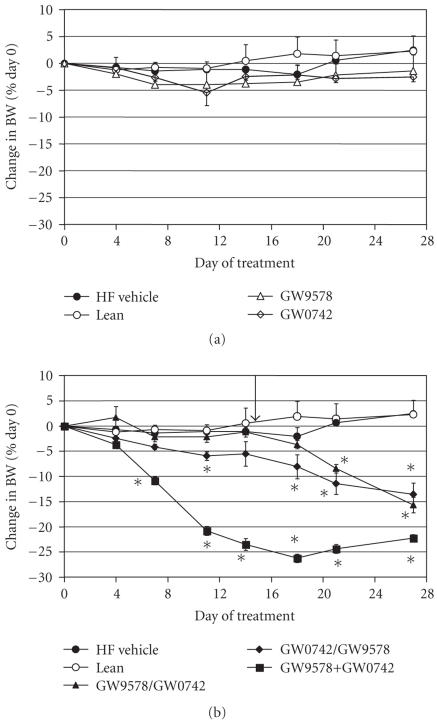
Effect of treatment with selective PPAR*α* and PPAR*δ* agonists on BW in lean and DIO AKR/J mice. (a) GW9578, a PPAR*α* agonist (1 mg/kg), GW0742, a PPAR*δ* agonist (30 mg/kg). (b) Filled triangle: GW9578 dosed for 14 days then combined with GW0742; filled diamond: GW0742 dosed for 14 days then combined with GW9578; filled square: GW9578 and GW0742 dosed together for 28 days. The arrow indicates the point at which the sequential combination of PPAR*α* and PPAR*δ* began. Data were analyzed by ANCOVA with repeated measures followed by Dunnett's post hoc test. Values were considered to be significant (*) when a value of *P* < .05 was achieved. *N* = 8–10 animals/group.

**Figure 2:**
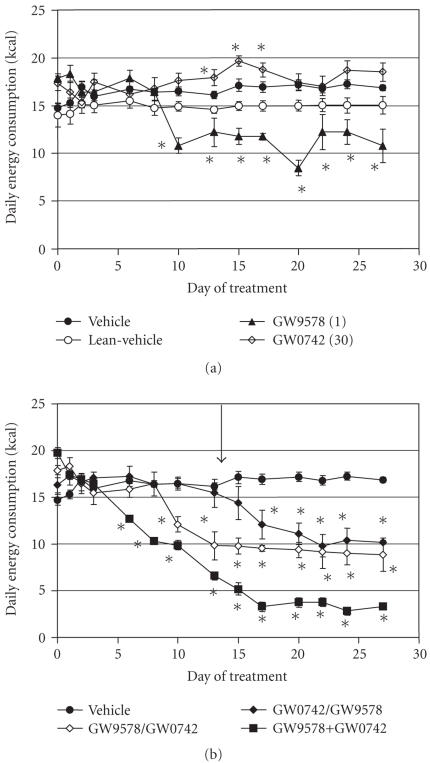
Effect of treatment with selective PPAR*α* and PPAR*δ* agonists on food consumption (kcal) in lean and DIO AKR/J mice. (a) GW9578, a PPAR*α* agonist (1 mg/kg), GW0742, a PPAR*δ* agonist (30 mg/kg). (b) Filled triangle: GW9578 dosed for 14 days then combined with GW0742; filled diamond: GW0742 dosed for 14 days then combined with GW9578; filled square: GW9578 and GW0742 dosed together for 28 days. The arrow indicates the point at which the sequential combination of PPAR*α* and PPAR*δ* began. Data were analyzed by ANCOVA with repeated measures followed by Dunnett's post hoc test. Values were considered to be significant (*) when a value of *P* < .05 was achieved. *N* = 8–10 animals/group.

The first experiment was designed to compare the effects of selective PPAR*α* (GW9578, 1 mg/kg) and PPAR*δ* (GW0742, 30 mg/kg) agonists, and the combination of the two agents, on BW, fat mass (FM), lean mass (LM), and food consumption. Data are shown in Figures 1, 2, and Table 2. Vehicle-treated mice weighed approximately 50 grams at initiation of the study and BW did not change during the study. While there was an initial weight loss trend, neither compound induced a sustained decrease in BW after 28 days of dosing (see Figure 1(a)).

On day 0, FM and LM (see Table 2) comprised 21.8 ± 1.6% (7.2 ± 0.6 grams) and 63.1 ± 1.3% (20.8 ± 0.4 grams) of total body weight, respectively, in lean mice. The remaining mass of each animal is composed of bone, free water (as cellular, interstitial and, blood volumes), and the contents of the gastrointestinal tract and bladder. In the DIO vehicle group, FM was nearly twice that of the lean mice (16.1 ± 1.4 grams; 39.5% of BW), but LM was similar (21.2 ± 0.2 grams; 53.1% of BW). FM and LM did not change in the DIO or lean vehicle groups in any of the experiments.

In spite of the fact that neither agent produced a significant decrease in BW, there was a slight decrease in FM after treatment with either the PPAR*α* or PPAR*δ* agonist while LM was unaffected (see Table 2, Experiment 1). Both agents produced a statistically significant increase in liver weight of nearly 1 gram that appears to have counterbalanced the change in fat mass resulting in unaltered BW.

Both the PPAR*α* and PPAR*δ* agonists affected food consumption. Compared to vehicle-treated animals, the PPAR*α* agonist reduced food consumption while the PPAR*δ* agonist produced a small but statistically significant increase in feeding (see Figure 2(a)). The effect of PPAR*α* activation on feeding did not occur until day 10, the same point when weight loss had reached a plateau and subsequently began to rebound.

A second goal of Experiment 1 was to examine the effects of PPAR*α* and PPAR*δ* in combination on BW, body mass, and food consumption. Minimal BW changes were observed with the PPAR*α* or PPAR*δ* agonists alone similar to Figure 1(a). At Day 14, the PPAR*α* agonist was added to the group dosed with PPAR*δ* alone or vice versa for an additional 14 days. Both conditions resulted in weight loss (see Figure 1(b)) greater than observed with either agent alone. The overall weight loss from either combination was approximately 15% which was commensurate to the decrease in FM. A third group of mice was dosed with a combination of the PPAR*α* and PPAR*δ* agonists for the entire 28- day period. This treatment resulted in a 22% reduction in BW that occurred by 14 days. Both final BW and FM were similar to that of lean controls.

Addition of the PPAR*δ* agonist to the PPAR*α* agonist dosing regimen at 14 days did not have a significant effect on food consumption (see Figure 2(b)). However, adding PPAR*α* to the dosing regimen of mice receiving the PPAR*δ* agonist reduced food consumption to the level seen with PPAR*α* agonist alone. Interestingly, simultaneous dosing from study outset with both the PPAR*α* and PPAR*δ* agonists reduced feeding to a greater extent then the sequential addition of the agents.

*Experiment 2. Effect of a PPAR*γ* agonist and rimonabant in obese AKR/J mice*

Where Experiment 1 focused on the effects of selective PPAR*α* and PPAR*δ* agonists, Experiment 2 was designed to examine the effect of GW7845, a selective PPAR*γ* agonist dosed at 3 mg/kg on BW, FM, LM, and food consumption. RIM, a CB-1R antagonist was used as a positive control for weight loss.

**Figure 3:**
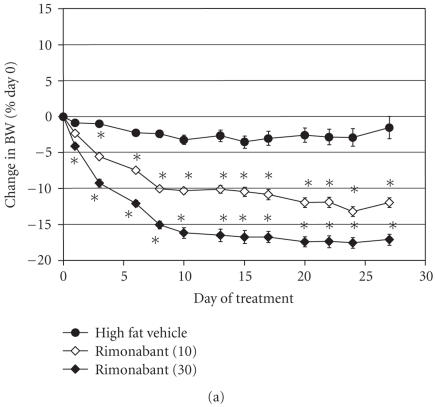

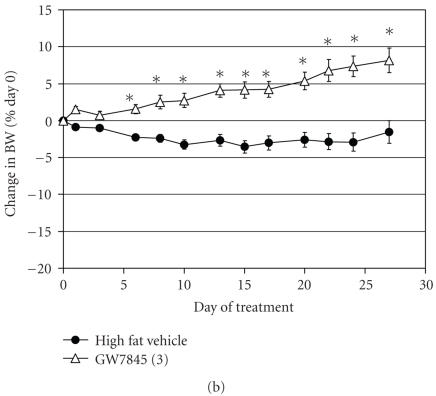
Effect of treatment with rimonabant or selective PPAR*γ* agonist on BW. (a) RIM (10 and 30 mg/kg). (b) GW7845, a selective PPAR*γ* agonist (3 mg/kg). Data were analyzed by ANCOVA with repeated measures followed by Dunnett's post hoc test. Values were considered to be significant (*) when the value of *P* < .05 was achieved. *N* = 8–10 animals/group.

Treatment with RIM at doses of 10 and 30 mg/kg produced significant, dose-related decreases of BW. At the highest dose, RIM reduced BW by 17% within the first 10 days of treatment (see Figure 3(a)) and the effect was maintained over the remainder of the study. RIM also decreased FM in a dose-dependent manner (see Table 2, Experiment 2). In contrast, the PPAR*γ* agonist produced a steady and consistent increase in BW over the course of the experiment (see Figure 3(b)). After 28 days, the weight of these animals had increased by almost 4 grams (8.6 ± 1.4% BW) and the mice were continuing to gain weight at 4 weeks. The PPAR*γ* agonist produced a significant increase in FM over the 28 days of the study accounting for much of the weight gain in these animals.

**Figure 4:**
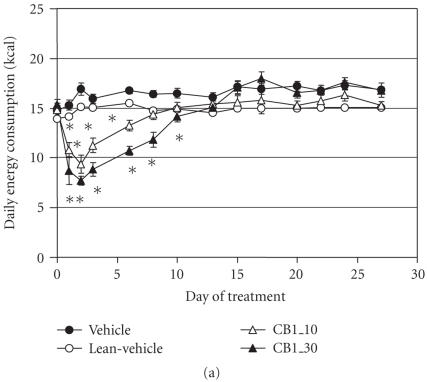

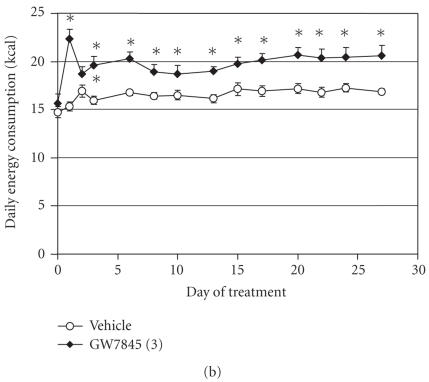
Effect of treatment with rimonabant or selective PPAR*γ* agonist on food consumption (kcal). (a) RIM (10 and 30 mg/kg). (b) GW7845, a selective PPAR*γ* agonist (3 mg/kg). Data were analyzed by ANCOVA with repeated measures followed by Dunnett's post hoc test. Values were considered to be significant (*) when the value of *P* < .05 was achieved. *N* = 8–10 animals/group.

RIM induced dose-related decreases in food consumption with the greatest suppression observed on day 3 (see Figure 4(a)). After day 3, food consumption suppression began to wane, eventually returning to control levels by day 10 and remained at that level for the duration of the study. In contrast to the effect of RIM, food consumption of animals dosed with the PPAR*γ* agonist increased 46% after only one day and remained elevated by more than 20% over the remaining treatment period (see Figure 4(b)).

*Experiments 3 and 4. Effect of PPARpan agonists in obese AKR/J mice*

**Figure 5:**
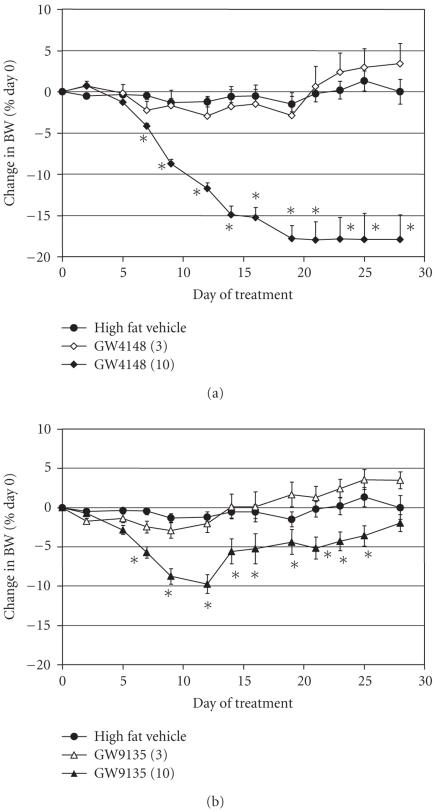
Effect of treatment with PPARpan agonists on BW. (a) GW4148 dosed at 3 and 10 mg/kg. (b) GW9135 dosed at 3 and 10 mg/kg. Data are expressed as mean ± SEM and were analyzed by ANCOVA with repeated measures followed by Dunnett's post hoc test. Values were considered to be significant (*) when the value of *P* < .05 was achieved. *N* = 8 animals/group.

Experiments 3 and 4 explore the effects of two PPARpan agonists with different selectivity profiles (see Figure 5, Table 1). GW4148, a potent activator of all three PPAR receptor subtypes, was used in Experiment 3. Dosed at 3 mg/kg, GW4148 did not induce weight loss. In contrast, a dose of 10 mg/kg significantly decreased BW by 18% after 19 days of dosing (see Figure 5(a)). This change mirrored the effects seen when PPAR*α* and PPAR*δ* were coadministered in Experiment 1. GW4148 also produced a significant decrease in FM that was commensurate with the reduction in BW.

GW9135 is a PPARpan compound with a different pattern of activation than GW9148, being very potent at PPAR*α* and PPAR*γ* and weaker at PPAR*δ*. Dosing GW9135 at 3 mg/kg had no effect on BW (see Figure 5(b)). Treatment with 10 mg/kg GW9135 reduced body weight 10% by day 8, however, the mice regained weight after that time and final BW was not significantly different from vehicle-treated animals at day 27 (see Figure 5(b)). This dose of GW9135 significantly reduced FM by 4 grams. Both GW4148 and GW9135 treatments increased liver weights by approximately 2.5 grams (see Table 2) which counterbalanced the final BW to some extent.

*Effect of PPAR agonists on serum chemistry*

Serum chemistry results are shown as group means in Table 3. None of the PPAR agonists tested in these experiments had a significant effect on blood glucose levels. The PPAR*α* and PPAR*δ* agonists alone significantly reduced circulating insulin (INS) levels. The combination of the two agents not only reduced insulin but also significantly reduced triglyceride (TG) and nonesterified fatty acids (NEFAs) and elevated total cholesterol (CHOL), high-density lipoprotein cholesterol (HDL-c), and *β*-Hydroxybutyric acid (*β*HBA). The selective PPAR*γ* agonist produced a significant reduction in circulating INS, TG, and NEFA levels. Both PPARpan agonists significantly reduced fed glucose, INS, NEFAs, and TG and increased total CHOL, HDL-c, and *β*HBA.

*Effect of PPAR agonists on ex vivo fatty acid oxidation*

**Figure 6:**
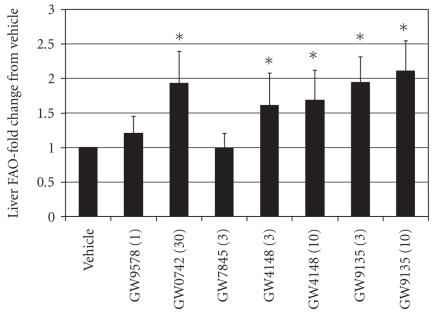
Effect of PPAR agonist treatment on fatty acid oxidation (FAO) in liver was assessed using a ^14^C capture method modified from Dohm et al. [28]. Data are expressed as fold change from vehicle control (mean ± SEM). *N* = 6 determinations/compound.

Changes in drug-induced fatty acid oxidation (FAO) were evaluated in mouse liver extracts from animals treated with compound for 28 days (see Figure 6). Activation of the PPAR*δ* agonist produced a 1.9-fold increase in FAO while the PPAR*γ* agonist and PPAR*α* agonist were not different from vehicle. The PPARpan agonists elicited responses similar to the PPAR*δ* agonist and this response most likely reflects the activity of PPARpan agonists at the PPAR*δ* receptor.

## 6. DISCUSSION

There is a critical medical need to develop effective strategies for long-term weight loss and weight maintenance although it is unlikely that any single therapy will yield maximal efficacy. Currently, the few therapies actually shown to be effective for weight loss include lifestyle modifications (diet and exercise), bariatric surgery, and pharmacological targets that modulate central pathways that regulate food intake [41]. PPARs are known to modulate enzymes involved in lipid metabolism and are expressed in many, if not all, metabolically active tissues including liver, heart, kidney, skeletal muscle, intestine, pancreas, and adipose tissue [42, 43]. This suggests that PPARs play a key role in energy metabolism and homeostasis that may ultimately affect body weight and body mass. In this report, we present data showing that potent and selective agonists of all three PPAR isoforms serve to modulate food intake and energy balance in DIO AKR/J mice.

Selective activators of PPAR*γ*, such as glitazones, have been successfully used to treat T2DM for nearly a decade. Treatment with rosiglitazone and pioglitazone induce body weight gain in mice [45, 46, 49], rats [44, 47–50], nonhuman primates [51, 52], and humans [53–55]. Weight gain is manifested as increased adiposity, total body water and plasma volume. In this report, mice treated with a potent and selective PPAR*γ* activator gained more weight than obese vehicle controls and the weight gain could be completely accounted for by increased fat mass which was equivalent to the increase in caloric intake. In addition to stimulation of food consumption, activation of PPAR*γ* promotes triglyceride accumulation by increasing expression of genes modulating adipogenesis [56–58], lipid transport [58, 59], storage [46, 60], and glucose homeostasis [61]. We also observed that GW7845 had no effect on FAO in mouse liver. In summary, PPAR*γ* agonism induces food consumption and energy storage without an effect on energy utilization resulting in net weight gain.

A number of studies have suggested that PPAR*δ* agonists regulate food intake, body weight, insulin sensitivity, and adiposity [8, 62–68]. Transgenic mice in which constitutively active PPAR*δ* is expressed in muscle are highly resistant to high-fat, diet-induced obesity [15]. Administration of GW501516, a selective PPAR*δ* agonist, promotes FAO and utilization, depleting lipid accumulation in adipocytes, skeletal muscle, and liver in DIO, ob/ob [68], and db/db mice [67].

Similarly, there are numerous studies that suggest that PPAR*α* can regulate food intake, body weight, and adiposity in rodents [69–74]. PPAR*α* has been shown to modulate target genes involved in uptake, activation, and degradation of fatty acids maintaining lipid homeostasis in liver, heart, and oxidative muscles [33, 75, 76]. It is possible that the combination of these mechanisms could result in reduction of body weight. Djouadi et al. [76] and Muoio et al. [33] have shown that the body weight of PPAR*α*-KO mice was greater than WT littermates, and that they became obese when fed a high fat diet, confirming the role of PPAR*α* receptors in modulating energy utilization and BW in rodents. In humans, fibrate treatment has not been associated with body weight loss (73), thus, the role of PPAR*α* agonism in human body weight regulation is unclear.

Neither PPAR*α* nor PPAR*δ* agonists had a sustained effect on body weight. While the increase in liver weights observed with both treatments counterbalanced the initial weight loss induced by these compounds, this change did not completely explain the rebound.

GW0742, the PPAR*δ* agonist, had a transient stimulatory effect on food intake from days 12–17 and it was during this time that the rebound increase in weight occurred. There was a significant increase in liver FAO induced by GW0742 after chronic dosing. The increase in food intake may have occurred in response to elevated energy expenditure, thus, an agent that only modulates energy expenditure did not induce significant weight loss in this model.

After 10 days of treatment with GW9578, the PPAR*α* agonist, a significant suppression of food intake was observed that persisted throughout the rest of the study. The timing of this effect coincided with the timing of the rebound in weight gain. Currently, we do not have an explanation for this phenomenon, yet it appears that chronic PPAR*α* agonism induces a metabolic compensation resulting in weight regain and the food intake suppression could be a counteracting mechanism. The effect on food consumption could be regulated centrally as PPAR*α* is expressed in low but detectable levels in mouse hypothalamus, a major center of appetite and satiety regulation. PPAR*α* could also modulate peripheral mechanisms that affect appetite or central response to lipid levels resulting from changes in FAO [12, 75]. While several reports have shown that PPAR*α* increased FAO, the measurement of this parameter at the end of the study indicated that there was only a modest alteration. We did observe weight loss during the first 10 days of the study without a change in food intake thus it is possible that there could have been induction of FAO during this time.

A combination study of PPAR*α* and PPAR*δ* agonists was performed to determine if greater weight loss could be achieved together than with either compound alone. After 2 weeks of dosing with either single agent, addition of the second agent further reduced body weight and fat mass, suggesting a synergistic effect of the two agents. Combination dosing of both agents for the entire 4 weeks of the study produced even greater reduction in body weight and fat mass. Interestingly, the suppression of food intake after addition of GW9578 to GW0742 and with the straight combination dosing occurred immediately as opposed to the 10-day delay observed with GW9578 alone. The immediate effect on food intake through PPAR*α*, increase in liver FAO from PPAR*δ*, and the initial induction of weight loss by PPAR*α* through a nonfood intake mechanism all account for the greater efficacy observed with the combination dosing from day 1 of treatment.

PPARpan agonists are a class of compounds that activate all three PPAR receptor subtypes and are currently being evaluated as antidiabetic agents. Compared to selective PPAR agonists, PPARpan ligands are expected to display unique characteristics as a result of ligand-activation profiles combining features of all three PPAR receptor subtypes, however, the effects are not simply the sum of the activities, but reflect a careful balance of lipid handling and energy. Both compounds used in this study are potent activators of all three isoforms but the potency ratio across the isoforms is different. GW4148 is an extremely potent agonist of murine PPAR*δ* (9 nM) and is 4-fold selective over PPAR*α* or PPAR*γ* receptors. In contrast, GW9135 is a potent agonist of murine PPAR*α* (13 nM) and is 18-fold and 50-fold selective over PPAR*γ* and PPAR*δ*, respectively. Other factors such as cofactor affinities contribute to the physiological behavior of each molecule.

GW9135 had little effect on overall weight loss, a pattern not different from PPAR*α* agonist treatment alone, where there was an initial decrease in weight followed by regain. This effect can be explained by the greater potency of the molecule at PPAR*α* and its weaker potency on PPAR*δ*. In contrast, GW4148, which is most potent at the PPAR*α* and PPAR*δ* receptors, behaved similarly to combination dosing of GW9578 and GW0742 producing significant weight loss at 10/mg/kg.

Contrary to the differential effects on body weight, both PPARpan agonists produced similar metabolic effects. Each compound reduced TG, NEFA, and circulating insulin levels, and elevated HDL-c and bHBA. A similar pattern was noted with the combination of GW9578 and GW0742, however, these two agents alone did not have significant effects on any parameter except insulin. The combination of PPAR*α* and PPAR*δ* activation results in a synergistic effect on serum chemistry parameters.

In summary, these studies demonstrate that PPARs are integrally involved in energy maintenance. The PPAR*α* and PPAR*δ* receptors are responsible for induction of weight loss in AKR/J mice through suppression of food intake and increased energy expenditure. Activation of PPAR*α* and PPAR*δ* receptors by PPARpan compounds may be expected to induce weight loss or provide weight maintenance while combining the beneficial insulin sensitization effects of a PPAR*γ* agonist.

## Figures and Tables

**Table 1:** Activation of murine PPAR receptors by PPAR agonists in cell-based transactivation assays. Compounds were assayed for agonist activity using the PPAR-GAL4 transactivation assay using an SPAP reporter transiently transfected in CV-1 cells as described in [25]. Data are mean ± SE of four or more independent experiments. The EC_50_ value was defined as the concentration of test compound that produced 50 ± 10% of the maximal reporter activity.

	Murine receptor activation (nM)					
	mPPAR*α*	%Max	mPPAR*δ*	%Max	mPPAR*γ*	%Max

GW9578	8.1	95	2344.2	76	2818.4	96
GW0742	8810.5	55	28.2	73	10000.0	67
GW7845	10770.9	30	10000.0	12	1.2	247
GW4148	41.8	114	9.4	134	37.3	88
GW9135	13.4	240	676.2	99	96.8	160

**Table 2:** Effect of treatment with PPAR agonists on body weight (BW), body composition (FM and LM), and liver weight (LW). Shown in the table are body weight (g) and fat and lean mass (g) values of each group. FM and LM were determined using qMR at the final day of the study (day 28). LW was obtained from terminal collection at the end of the experiment. *N* = 8 mice/group. Data are expressed as mean +/− SEM. Doses are in mg/kg. Data were analyzed by two-way ANOVA with repeated measures followed by post hoc *t*-test. Data achieved significance when *P* < .05(*).

	Treatment	BW day 0 (grams)	BW day 28 (grams)	Fat mass day 28 (grams)	Lean mass day 28 (grams)	Liver weight (grams)

Experiment 1	Lean vehicle	36.6 ± 0.8	37.0 ± 1.0	8.4 ± 1.1	23.2 ± 0.4	1.8 ± 0.1
	DIO vehicle	50.4 ± 1.3	52.1 ± 1.8	20.8 ± 1.3	29.3 ± 0.7	2.0 ± 0.2
	GW9578 (1)	52.1 ± 1.1	51.3 ± 1.6	18.4 ± 0.9	30.9 ± 0.8	2.6 ± 0.1*
	GW0742 (30)	51.6 ± 1.1	50.2 ± 1.2	18.4 ± 0.7*	29.7 ± 0.7	3.2 ± 0.1*
	GW9578 + GW4148 (after week 2)	49.0 ± 0.6	41.3 ± 1.2*	10.2 ± 0.9*	29.1 ± 0.5	4.3 ± 0.2*
	GW4148 + GW9578 (after week 2)	49.5 ± 1.0	42.7 ± 0.8*	11.0 ± 0.8*	29.5 ± 0.9	4.3 ± 0.1*
	GW9578 and GW4148 4 weeks	50.5 ± 0.9	39.3 ± 0.9*	9.1 ± 0.4*	28.4 ± 0.7	4.9 ± 0.1*

Experiment 2	DIO vehicle	50.8 ± 0.4	49.2 ± 0.5	20.7 ± 0.8	25.4 ± 1.0	1.9 ± 0.1
	RIM (10)	50.6 ± 0.8	44.1 ± 1.3*	15.0 ± 0.9	25.2 ± 0.8	2.0 ± 0.1
	RIM (30)	51.0 ± 0.7	41.8 ± 0.5*	11.6 ± 0.3	26.5 ± 0.5	2.0 ± 0.1
	GW7845 (3)	50.9 ± 1.2	54.6 ± 1.7*	23.5 ± 1.3	28.7 ± 0.6	2.0 ± 0.1

Experiment 3	DIO vehicle	40.8 ± 1.3	44.9 ± 1.6	17.6 ± 1.6	23.5 ± 0.5	1.9 ± 0.1
	GW4148 (3)	40.8 ± 1.2	42.0 ± 0.9	13.5 ± 0.9*	23.7 ± 0.3	2.9 ± 0.1*
	GW4148 (10)	40.7 ± 1.5	36.6 ± 0.9*	10.3 ± 0.6*	22.3 ± 0.5	3.4 ± 0.1*

Experiment 4	Lean vehicle	33.7 ± 0.7	34.4 ± 0.8	8.4 ± 0.8	22.0 ± 0.6	1.7 ± 0.1
	DIO vehicle	40.6 ± 1.9	43.1 ± 1.6	16.5 ± 1.1	23.4 ± 0.6	1.9 ± 0.1
	GW9135 (3)	41.1 ± 1.8	42.5 ± 1.9	14.9 ± 1.5	24.1 ± 0.3	3.0 ± 0.2*
	GW9135 (10)	41.2 ± 1.5	40.3 ± 1.2	12.3 ± 0.8*	23.0 ± 0.4	3.3 ± 0.1*

**Table 3:** Group means of clinical chemistry results of DIO-AKR mice. Terminal blood samples were obtained at the end of treatment. Serum levels of analytes were determined using an Olympus AU640 clinical chemistry analyzer and analyzed by a two-way analysis of variance with repeated measures model (ANOVA) followed by Dunnett's post hoc test. Values were considered to be significant (*) when the value of *P* < .05 was achieved.

	Treatment	Glucose (mg/dL)	Insulin (ng/mL)	Triglyceride (mg/dL)	NEFA (mEq/L)	Cholesterol (mg/dL)	HDL-C (mg/dL)	bHBA(mg/dL)

Experiment 1	Lean vehicle	211.3 ± 7.8	1.7 ± 1.4	194.7 ± 0.7	0.7 ± 0.03	89.5 ± 1.6	57.2 ± 1.1	2.1 ± 0.2
	DIO vehicle	241.2 ± 7.4	11.2 ± 2.1	154.5 ± 8.1	0.8 ± 0.02	188.7 ± 12.5	131.8 ± 4.5	2.0 ± 0.2
	GW9578 (1)	248.0 ± 16.6	3.1 ± 1.6*	168.3 ± 15.4	0.9 ± 0.04	117.1 ± 4.1	86.6 ± 2.7	3.2 ± 1.1
	GW0742 (30)	253.3 ± 8.9	2.2 ± 1.2*	174.9 ± 10.3	0.9 ± 0.02	171.9 ± 2.9	118.4 ± 1.6	3.8 ± 0.3*
	GW9578 + GW0742 after week 2	211.5 ± 14.2	1.9 ± 0.9*	97.5 ± 5.8*	0.6 ± 0.02*	207.5 ± 4.5*	131.8 ± 4.5	7.0 ± 0.5*
	GW4148 + GW0742 after week 2	157.4 ± 2.4*	2.9 ± 1.1*	100.0 ± 8.9*	0.6 ± 0.04*	210.1 ± 8.5*	132.5 ± 4.7	7.0 ± 1.4*
	GW9578 and GW0742 4 weeks	199.1 ± 1.6*	2.6 ± 0.8*	128.1 ± 9.0*	0.8 ± 0.04	234.4 ± 5.5*	141.8 ± 2.3	6.9 ± 0.8*

Experiment 2	DIO vehicle	225.4 ± 11.7	10.2 ± 0.5	152.3 ± 0.8	0.7 ± 0.8	114.9 ± 2.9	81.8 ± 1.6	1.6 ± 0.2
	RIM (10)	230.1 ± 16.6	7.2 ± 2.5*	181.4 ± 36.1	0.8 ± 0.05	147.3 ± 9.1*	95.1 ± 4.6*	2.5 ± 0.2*
	RIM (30)	215.3 ± 13.0	3.4 ± 0.7*	159.7 ± 16.1	0.9 ± 0.02	143.4 ± 4.0*	116.9 ± 2.6*	1.8 ± 0.2
	GW7845 (3)	222.3 ± 9.4	2.1 ± 0.4*	112.3 ± 5.7*	0.6 ± 0.03*	106.1 ± 4.2	65.0 ± 0.9*	1.5 ± 0.1

Experiment 3	DIO vehicle	234.0 ± 9.4	6.7 ± 1.9	163.5 ± 16.2	0.8 ± 0.07	116.1 ± 5.9	91.1 ± 3.7	2.9 ± 0.2
	GW4148 (3)	233.3 ± 14.6	1.8 ± 0.3*	62.6 ± 4.1*	0.5 ± 0.03*	178.9 ± 3.7*	52.9 ± 0.1*	5.1 ± 0.9*
	GW4148 (10)	214.0 ± 10.9*	1.3 ± 0.3*	48.4 ± 3.6*	0.5 ± 0.04*	192.6 ± 6.8*	131.1 ± 3.6*	6.5 ± 1.1*

Experiment 4	Lean vehicle	189.5 ± 8.6	1.2 ± 0.2	220.5 ± 14.0	0.7 ± 0.04	70.0 ± 1.9	51.7 ± 1.4	1.6 ± 0.1
	DIO Vehicle	196.0 ± 11.1	11.2 ± 2.1	277.6 ± 25.2	1.5 ± 0.10	122.8 ± 4.7	106.0 ± 2.8	3.7 ± 0.6
	GW9135 (3)	215.0 ± 14.1	2.9 ± 0.5*	113.8 ± 9.9*	0.8 ± 0.04*	188.9 ± 4.9*	149.4 ± 2.4*	4.4 ± 0.6*
	GW9135 (10)	196.1 ± 5.6	1.4 ± 0.4*	58.8 ± 2.4*	0.6 ± 0.02*	183.8 ± 5.2*	141.9 ± 2.8*	4.3 ± 0.4*

## ABBREVIATIONS

PPAR: Peroxisome proliferator-activated receptor
RIM: Rimonabant
CB1-R: Cannabinoid 1-receptor
d: Day
BW: Body weight
FM: Fat mass
LM: Lean mass
LW: Liver weight
qMR: Quantitative magnetic resonance
FAO: Fatty acid oxidation
GLU: Glucose
INS: Insulin
TG: Triglyceride
NEFA: Nonesterified fatty acid
CHOL: Cholesterol
HDLc: High-density lipoprotein cholesterol
*β*HBA: *β*-hydroxybutyric acid
